# *Astragalus Polysaccharides* Protects Thapsigargin-induced Endoplasmic Reticulum Stress in HT29 Cells

**DOI:** 10.1515/biol-2019-0055

**Published:** 2019-12-31

**Authors:** Lie Zheng, Ya-Li Zhang, Xuan Chen, De-Liang Chen, Yan-Cheng Dai, Zhi-Peng Tang

**Affiliations:** 1 Institute of Digestive Diseases, LongHua Hospital, Shanghai University of Traditional Chinese Medicine, 725 Wanping Road, Shanghai 200032, China; 2 Department of Gastroenterology, Shanghai Traditional Chinese Medicine-Integrated Hospital, Shanghai University of Traditional Chinese Medicine, 230 Baoding Road, Shanghai 200082, China; 3 Department of Gastroenterology, Shaanxi Hospital of Traditional Chinese Medicine, Xi’an 710003, Shaanxi, Xi’an, China; 4 Shanghai University of Traditional Chinese Medicine, Shanghai 201203, China; 5 Department of Tuina, Affiliated Hospital of Nanjing University of Chinese Medicine, Nanjing 210029, China

**Keywords:** *Astragalus polysaccharides*, Thapsigargin, ER stress, HT29 cells, PERK-eIF2a signaling

## Abstract

**Aim:**

This study investigates the effect of *astragalus polysaccharides* (APS) in protecting against thapsigargin-induced endoplasmic reticulum (ER) stress in HT29 cells by suppressing the PERK-eIF2a signaling pathway.

**Methods:**

HT29 cells were induced by thapsigargin for 12 hours, then treated with APS for 24 hours, and the gene expressions of GRP78, CHOP and eIF2a were quantified by reverse transcription quantitative polymerase chain reaction (RT-qPCR). The expression of GRP78, CHOP, PERK, *p*-PERK, eIF2a, and *p*-eIF2a were detected by Western blot.

**Results:**

The ER stress caused by thapsigargin strongly up-regulated the expression of GRP78 and CHOP in HT29 cells, which activated the PERK-eIF2a pathway. There was an increase in PERK phosphorylation, and induction of eIF2a in HT29 cells. Thapsigargin caused significant ER expansion in HT29 cells due to the 12-hour ER stress. Importantly, *Astragalus polysaccharide* significantly inhibited the phosphorylation of PERK and eIF2a, which reduced the mRNA levels of GRP78, CHOP, PERK and eIF2a, and inhibited the ER expansion in HT29 cells after 24 hours of treatment.

**Conclusion:**

The results indicate that APS reduces the expression of GRP78 and CHOP in HT29 cells, at least in part, by preventing the activation of the PERK-eIF2a signaling pathway.

## 1 Background

The endoplasmic reticulum (ER) is the major site for protein synthesis and folding. It serves many functions, this includes (1) protein folding, glycosylation and sorting; (2) the synthesis of cholesterol and other lipids; (3) the maintenance of Ca^2+^ homeostasis. The disruption of any of these processes causes ER stress, which results in disturbed protein synthesis, folding, transport, or degradation, as well as Ca^2+^ overload [1,2]. As a result, cells have evolved various protective strategies for overcoming ER stress, which have been collectively termed as the unfolded protein response (UPR). The UPR is mediated by ER stress transducers, one major stress transducer of the UPR is PEKR-like endoplasmic reticulum kinase (PERK) [3,4]. The transducer senses the presence of protein displayed in the ER lumen, as well as the transduction signal of the cytoplasm or nucleus. The activation of PERK results in the phosphorylation of eukaryotic initiation factor 2 (eIF2), preventing the assembly of 80S ribosomes, and protein synthesis [5,6]. Although the activation of UPR may maintain the survival of cells, the adaptive response of UPR cannot eliminate the ER stress. Cells cannot restore ER homeostasis under severe or persistent ER stress, leading to activation of the apoptosis signaling pathway.

*Astragalus* is a traditional Chinese medicine that has been deeply studied and widely used in clinical practice [7,8]. The main component of *Astragalus* is *Astragalus polysaccharide* (APS). APS has been shown to possess a variety of biological activities, including anti-oxidative, immunomodulatory, anti-tumor, anti-inflammatory and anti-viral activities [9]. In addition to the above-mentioned activities, it has been shown to prevent the activation of the PERK-eIF2a signaling pathway. This study confirms the aforementioned pathway relationships and the usefulness of traditional APS herbal treatment for the described purposes. In this study we use HT29 human colon cancer cell line. These cells have similar biological characteristics with intestinal epithelial cells (IECs) and are easily cultured. These cells are an internationally recognized model of ulcerative colitis inflammatory cells. We demonstrate that APS can protect against ER stress caused by thapsigargin in HT29 cells, via suppression of the PERK-eIF2a signaling pathway [10,11].

## 2 Materials and Methods

### 2.1 General reagents

APS was purchased from Shanghai Yuanye Biotechnology Co., Ltd. (Shanghai, China), and the HT29 cells were obtained from Shanghai Life Sciences Research Institute (Shanghai, China).

### 2.2 HT29 cells grouping

After conventional culture of HT29 cells, the cells were divided into four groups according to the experimental design (1). Control group: cells no drug treatment; (2) Model group: Tg-induced HT29 cells were used to establish ER stress model. (3) Astragalus polysaccharide low concentration group (1 μg/mL+1 μmol/L of Tg, AP-L group); (4) high concentration of Astragalus polysaccharide group (10 μg/mL+ 1 μmol/L of Tg, AP-H group).

### 2.3 Cell culture

The HT29 cells were cultured with McCoys 5A (Sigma, St. Louis, MO, USA) and 2 mM glutamine (Sigma, St. Louis, MO, USA), 100 units/mL penicillin. For basic cell culture maintenance, 100 mg/mL amphotericin and 10% fetal bovine serum (FBS, Sigma, USA) were used. Each culture dish was coated with collagen (0.012 mg/mL, Sigma, St. Louis, MO, USA) overnight before use. Cells were transferred to differentiation medium (McCoys 5A supplemented with 10% FBS) and incubated at a density of 0.75×10^5^ cells/mL in a humidified atmosphere (95% air and 5% CO_2_) for five days. The differentiation medium was replaced every other day. The differentiated HT29 cells were cultured at 37.5°C in a humidified atmosphere (95% air and 5% CO_2_) in McCoys 5A medium, supplemented with 2 mM glutamine (Sigma, St. Louis, MO, USA), 100 units/mL penicillin, 100 mg/mL streptomycin, and 10% FBS. After 3-5 days, and when cell growth reached 80% confluence at the bottom of the culture dish, these cells were subdivided into two or three generations.

### 2.4 Cell viability assay

The HT29 cells were plated at a density of 5×10^4^ cells/well in 96-well plates, and cell viability was assessed using Cell Counter Kit-8 (CCK-8) assay (Dojindo Laboratories, Kyushu, Japan), the assay was performed according to the manufacturer’s instructions. Briefly, cells were incubated at 37.5°C with or without thapsigargin for 24 hours. Next, 10 mL of CCK-8 solution was added to each well, and these cells were incubated for an additional 1.5 hours. Absorbance was calculated using an automatic reader (Bio-Tec, CA, USA), and was performed three times per assay. The assay was repeated for three times.

### 2.5 Protein extraction and Western blot analysis

Western blot analysis was performed as described in a previous study [12]. HT29 cells were treated with different APS concentrations (0, 1, 10 μg/mL) at different time points (12, 24 and 48 hours). The control cells were only treated with McCoys 5A. After the indicated treatments, approximately 10^5^ cells were washed three times with cold phosphate-buffered saline (PBS) and lysed at 48°C for 30 minutes using cell lysis buffer containing 50 mM Tris (pH 8.0), 150 mM NaCl, 1 mM ethylenediaminetetraacetic acid, 1% sodium deoxycholate, 1% Nonidet P-40 (NP-40) and 1× protease inhibitor cocktail (Roche Diagnostics, Winterthur, Switzerland). Cell lysates were centrifuged at 13,000 rpm for 15 minutes at 4°C. Afterwards, the supernatant was transferred to new tubes, and the protein concentration was determined using a Bradford protein assay kit (Bio-Rad, Hartford, VT, USA). Next, the sample buffer was added to the cytosolic extracts, boiled for 10 minutes, and cooled in an ice bath. Equal volumes of supernatant from each sample were separated by 10% sodium dodecyl sulfate (SDS)-polyacrylamide gel electrophoresis and electro-transferred to polyvinylidene difluoride membranes (Millipore, Bedford, MA, USA). Membranes were then blocked with 5% skim milk for two hours at room temperature and incubated overnight at 4°C with antibodies against glucose-regulated protein 78 (GRP78), CHOP, PERK, *p*-PERK, eIF2a, *p*-eIF2a, (1:1,000; Cell Signaling, MA, USA). Afterwards, the membranes were incubated with horseradish peroxidase-conjugated anti-rabbit IgG (1:2,000; Cell Signaling, MA, USA) for two hours, and visualized using enhanced chemiluminescence (Pierce Company, Woburn, MA, USA). Each experiment was performed in triplicate.

### 2.6 RNA isolation and quantitative polymerase chain reaction (qPCR)

The tissue samples were frozen and separated in mechanical RNA buffer, and total RNA was extracted using RNA prep Pure Cell/Bacterial Kit (Tiangen, China) according to the manufacturer’s protocol. An Eppendorf PCR system, which involved the Quanti Fast SYBR Green PCR Master Mix (TOYOBO, Osaka, Japan), primers (1 mM, Table 1) and 1 μL of cDNA in a 20-μL reaction mixture, was used to carry out the reverse transcription quantitative polymerase chain reaction (RT-qPCR). The cycle conditions used were as follows: Holding stage - 95°C for 30 seconds, Cycling stage - 95°C for five seconds, 55°C for 10 seconds, and 72°C for 15 seconds for 40 cycles. Immediately after the amplification, the melt curve protocol was used to ensure that minimization occurred in the primer and the other nonspecific products. The ^ΔΔ^Ct method was used to analyze the expression of the target genes (Table 1). The assay was repeated three times.

**Table 1:** List of primers used in this study

Number	Gene		Primer sequence	Primer length
1	GRP78	Forward-	5’-GTCCTATGTCGCCTTCACTCC-3’	21
		Reverse-	5’- GCACAGACGGGTCATTCCAC-3’	20
2	CHOP	Forward-	5’- CTGGACCGCTTGGGTAACTC-3’	20
		Reverse-	5’- GGCTATTGCTCATCATGGCTAG-3’	22
3	PERK	Forward-	5’- CTCGGGAAAAGGTAATGCG-3’	19
		Reverse-	5’- ATCCATCTTTTCTTGCCACTTC-3’	22
4	eIF2a	Forward-	5’- GATTGAGGAAAAGAGGGGTGTG-3’	22
		Reverse-	5’- TTTGGCTTCCATTTCTTCTGC-3’	21
5	β-actin	Forward-	5’-TGACGTGGACATCCGCAAAG-3’	20
		Reverse-	5’-CTGGAAGGTGGACAGCGAGG-3’	20

### 2.7 Electron microscopy

Electron microscopy was performed, as described previously [13]. Followed by these steps: (1) fixation: 2.5% glutaraldehyde and phosphoric acid buffer was used to prepare the fixed solution, and the fixed time was two hours; Cells were rinsed with 0.1M phosphate bleach solution three times, for 15 minutes each time. Then cells were treated with 1% osmium acid fixing solution for three hours, and then treated with 0.1M phosphoric acid bleaching solution three times, each time for 15 minutes. The samples were successively treated with the following reagents: first, immerse in 50% ethanol for 15 minutes; soaked in 70% ethanol for 15 minutes, soaked in 90% ethanol for 15 minutes and soaked in mixed solution of 90% ethanol +90% acetone (1:1) for 15 minutes. Then cells were soaked in 90% acetone for 15 minutes (in 4℃ refrigerator); Finally, cells were soaked in 100% acetone three times, each time for15 minutes (room temperature); (3) embedding method: first, cells were soaked in pure acetone + embedding solution (2:1) at room temperature for four hours, that is, 240 minutes, then soaked in pure acetone + embedded solution (1:2) overnight at room temperature, next soaked in the embedded solution at 37℃ for three hours, that is, 180 minutes. Curing: first, samples were left standing overnight in the oven at 37℃, then placed in the oven at 45℃ for 12 hours. Finally, they were placed in the oven at 60℃ for 24 hours. 5] slice: samples were sliced using an ultra-thin slicer to make continuous sections with a thickness of 50-60 nm; 6. Staining: double staining was performed with 3% uranium acetate and lead citrate; Ultrastructure of ER of epithelial cells in the colon was observed by using electron microscopy. The HT29 cells were treated with different APS concentrations (0, 1 and 10 μg/mL) for 24 hours. Then, cells were washed twice with PBS, and fixed in 4% glutaraldehyde. Next, cells were fixed with 1% OsO4, stepwise dehydrated in increasing concentrations of ethanol, and embedded in Epon 812 epoxy resin. Then, ultrathin sections were localized and viewed using a transmission electron microscope (JEM-1230; JEOL Ltd., Japan). Each sample was analyzed in triplicate.

### 2.8 Statistical analysis

SPSS 21.0 (SPSS, Chicago, IL, USA) was applied to analyze the research results. Measurement data were evaluated using *t*-test, while GraphPad Prism 5.0 (Graph Pad Software Inc., San Diego, CA, USA) was used for the generation of histograms. *P*<0.05 were considered statistically significant.

## 3 Results

### 3.1 Morphological observation of HT29 cell lines

**Figure 1:**
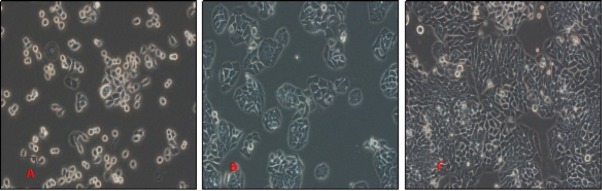
Growth of HT29 cells at different time points under the light microscopy at 400x magnification A: 24h, B:48h, C:72h

The HT29 cells were observed to have an irregular or spindle shape. After 24 hours of propagation, some of these cells attached on the wall, while most of these dispersed in a single cell state. After culture for 48 hours, it was clear that a large number of cells attached on the walls, had protrusions, and merged into each other forming clumps. At 72 hours of culture, these cells completely adhered to the wall, and were fused into a flaky shape (Figure 1).

### 3.2 Effects of thapsigargin on the activity of HT29 cells

**Figure 2:**
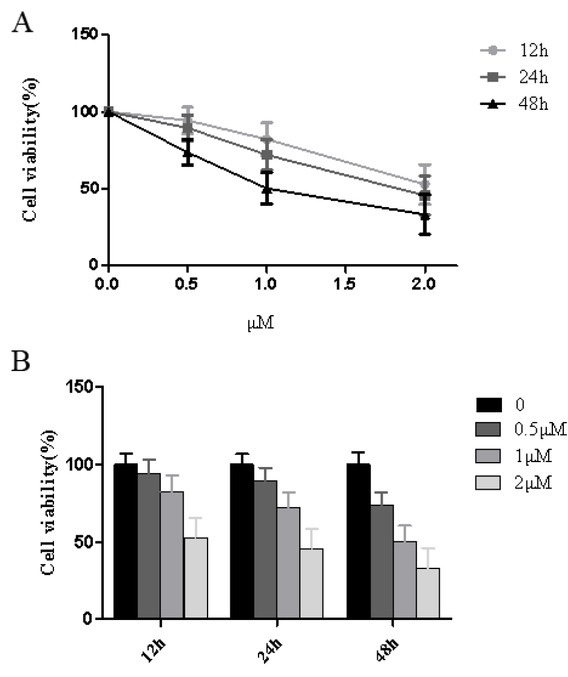
Effects of Thapsigargin treatment on cell viability of HT29 cells at different time points.

The thapsigargin concentrations used were 0.5, 1.0 and 2.0 μmol/L. At the same time, HT29 cells were assigned into the control group, and viable cells were measured at 12, 24 and 48 hours using the CCK-8 assay. The respective results are presented in Figure 2. HT29 cells treated with 0.5 μmol/L thapsigargin showed no significant difference to that of the control group at 12 hours (*P*>0.05). At 24 and 48 hours, the viability of all cells significantly decreased (*P*<0.05). Compared with cell viability at 24 hours, cell viability significantly decreased at 48 hours, cell viability was 73.65 ± 16.31%, and the difference was statistically significant (*P*<0.01). Similarly, treatment with thapsigargin concentration was 1 and 2 μmol/L, the cell viability decreased with time (*P*<0.05 or *P*<0.01), indicating that the thapsigargin toxicity was severe. The average cell viability was 72.07 ± 16.03% when treated with a concentration of 1 μmol/L for 24 hours. Thus, it was considered appropriate to select 1 μmol/L of thapsigargin for 12 hours for future experiments which is consistent with other studies (Figure 2).

### 3.3 Thapsigargin -induced ER stress response in HT29 cells

**Figure 3:**
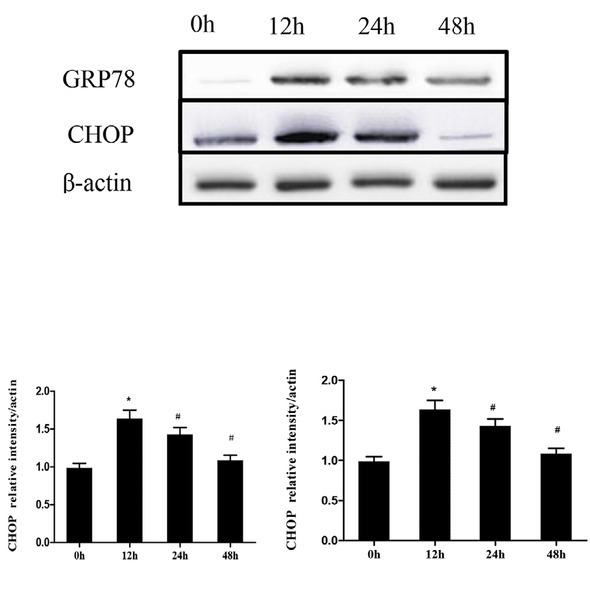
Western blot of ER stress markers following Thapsigargin treatment in HT29 cells

According to the results of the cell viability experiment, the cell viability was 82.23 ± 10.65% at 12 hours after the treatment of HT29 cells with 1 μM thapsigargin. This time point was chosen as the optimal time for ER membrane pressure, which was further confirmed by western blot of the ER marker, GRP78. Thapsigargin induced the expression of GRP78 and CHOP, which are two markers of ER stress in HT29 cells. At 12 hours, thapsigargin significantly increased expression of GRP78 and CHOP while at 24 and 48 hours, GRP78 and CHOP expression was reduced (*P*<0.05). At 48 hours there was a large reduction of CHOP expression (*P*<0.01). Over time, the expression of ER stress chaperone molecules slightly decreased, however, difference was statistically significant (*P*<0.05), when compared with that at 12 hours. Therefore, in combination with the results of the previous cell viability, the selected optimal concentration and time for the ER stress model of HT29 cells was 12 hours under 1 μM thapsigargin treatment. We demonstrated that thapsigargin causes significant ER stress in HT29 cells (Figure 3).

### 3.4 Astragalus polysaccharides suppressed the PERK-eIF2a pathway in HT29 cells

**Figure 4:**
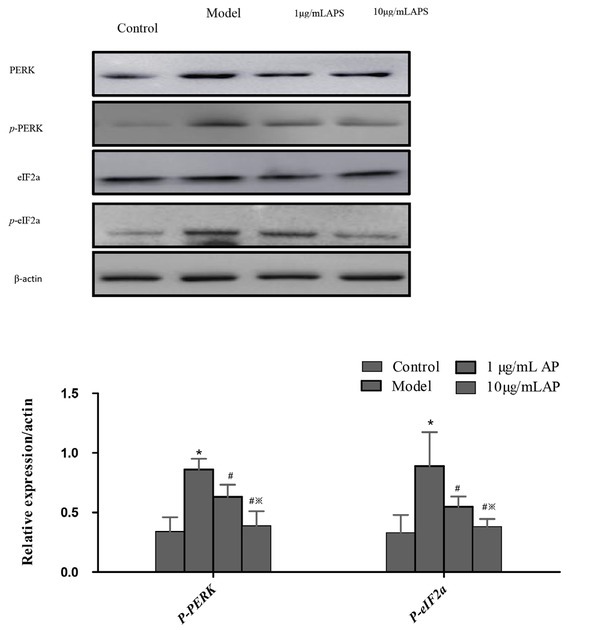
Western blot of proteins involved in the UPR pathway following APS treatment in HT29 cells.

The UPR is an important cell mechanism that responds to ER stress. We next undertook the analysis of a number of proteins involved in the UPR molecular signal transduction, PERK, *p*-PERK, eIF2a, and *p*-eIF2a. eIF2a is the downstream molecule of PERK, and the active domain of PERK is homologous with eIF2a kinases. Phosphorylated PERK initiates eIF2a phosphorylation. The treatment of HT29 cells with 1 or 10 μg/mL APS suppressed *p*-PERK and *p*-eIF2a levels; furthermore, a marked reduction in *p*-eIF2a was observed after 24 hours at a concentration of 10 μg/mL. Then, protein expressions after APS exposure declined to normal levels. We conclude that APS effectively suppressed the UPR and PERK-eIF2a pathway in HT29 cells (Figure 4).

### 3.5 Astragalus polysaccharides reduce the mRNA levels of GRP78, CHOP, PERK and eIF2a

**Figure 5:**
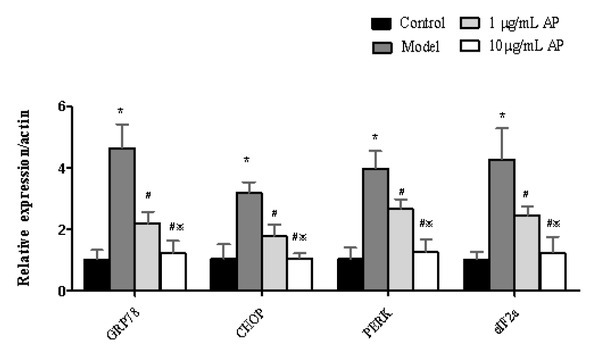
Astragalus polysaccharides reduce mRNA levels of GRP78, CHOP, PERK, and eIF2a

The expression of GRP78, CHOP, PERK and eIF2a were measured by RT-PCR in HT29 cells (Figure 5). An increase in this expression was detected, when compared to the control group. In contrast, with 1 or 10 μg/mL APS treatment there was a reduction in the expression of GRP78, CHOP, PERK and eIF2a (*P*<0.05).

### 3.6 Astragalus polysaccharides treatment disrupts the ER ultrastructure of HT29 cells

**Figure 6:**
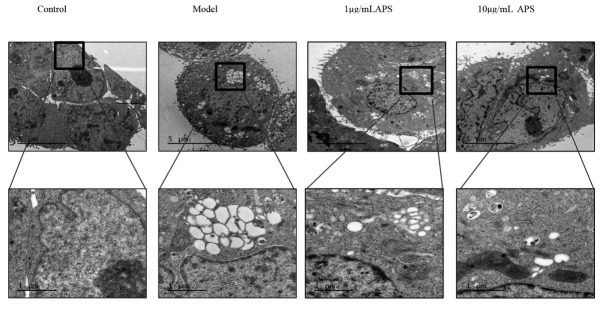
Astragalus polysaccharides treatment disrupts the ER ultrastructure of HT29 cells

The thapsigargin-induced ER stress that lasted for 12 hours resulted in obvious ER dilation in HT29 cells. The ER dilation was assessed by measuring the width of the largest lumen in each cell, and it was found that the ER ultrastructure had a normal flattened appearance (Figure 6). The treatment of cells with 1 or 10 μg/mL APS had significant effects on the dilated ER ultrastructure, thus 1 or 10 μg/mL APS suppressed the ER dilation. Furthermore, the widths of the ER lumen in HT29 cells treated with 1 or 10 μg/mL APS were significantly lower, when compared to that of the model group (*P*<0.05).

## 4 Discussion

The ER is a membranous network that provides a specialized environment for processing and folding newly synthesized proteins [14]. As metabolic demands increase, which perturb the protein folding in the ER, the same occurs in the workload of this protein factory, which is collectively called, ER stress. ER stress has been recently revealed to play a pivotal role in the pathogenesis of various diseases [15]. Since HT29 cells have a well-developed ER structure, these cells are appropriate for the study of colon-related diseases. Sustained ER stress is associated with increased apoptosis and death. We sought to determine whether APS is able to act on the PERK-eIF2a signaling pathway to suppress ER stress in HT29 cells [16].

ER stress in intestinal epithelial cells is one of the key mechanisms in the pathogenesis of ulcerative colitis. Chinese medicines have previously been shown to be effective systemic treatments for ulcerative colitis.

Furthermore, previous studies have suggested that the anti-inflammatory and immune-regulating function of certain Chinese medicines is associated with their ability to inhibit ER stress [18,19]. A traditional Chinese medicine, *Astragalus*, with APS as the main biologically active ingredient has been shown to boost immune function and suppress inflammation [17]. This is through suppressing ER stress in chronic diseases, such as ulcerative colitis and type-2 diabetes [23].

APS has previously been shown to protect cells by inhibiting oxidative stress-mediated apoptosis [22] and thus we hypothesized that APS may be a potential therapeutic for ulcerative colitis [20,21]. The present study demonstrates that APS can inhibit ER stress effects. Furthermore, APS effectively decreased the expression of *p*-PERK and *p*-eIF2a in HT29 cells. The activated UPR comprises of signaling pathways that induce ER stress, increase protein degradation and block new protein synthesis, and may subsequently result in apoptosis. We have shown the inhibitory effects of APS on the PERK-eIF2a signaling pathway and identified the part of its mechanism of attenuating ER stress in HT29 cells. Our study proposes that APS may be used to inhibit the expression of GRP78 and CHOP [24,25]. GRP78, also known as BiP, is the central regulator of the UPR, which has been widely used as an ER stress marker. Under normal conditions, GRP78 forms an inactive complex at the ER membrane, which comprises of three key UPR sensors: PERK, inositol-requiring enzyme 1 (IRE1), and activating transcription factor 6 (ATF6) [26]. When ER stress occurs, GRP78 is released from PERK, IRE1 and ATF-6, and binds to misfolded proteins, thereby activating the UPR. Consequently, the PERK, IRE1 and ATF6 pathways have been considered to be the three main pathways that mediate UPR signaling. The release of GRP78 allows PERK to dimerize and promote the phosphorylation of eIF2a, which in turn suppresses global mRNA translation to protect cells against ER stress [27]. The effects of APS on GRP78 and CHOP expression, as well as the activation of the UPR in HT29 cells, were examined. The expression of the following key UPR signal transduction molecules was analyzed: PERK, *p*-PERK, eIF2a, and *p*-eIF2a [28]. The treatment of HT29 cells with 1 or 10 μg/mL APS suppressed *p*-PERK and *p*-eIF2a levels. Furthermore, a marked reduction in *p*-eIF2a was observed after 24 hours of 10 μg/mL APS exposure, however, this subsequently declined to normal levels. This shows that APS can effectively suppress the UPR and the PERK-eIF2a pathway in HT29 cells. Next, the effects of APS on ER morphology in HT29 cells were evaluated using transmission electron microscopy [29,30]. The thapsigargin-induced ER stress for 12 hours resulted in obvious ER dilation in HT29 cells. The ER dilation was assessed by measuring the width of the largest lumen in each cell, and it was found that their ER ultrastructure had a normal flattened appearance [31]. The treatment of cells with 1 or 10 μg/mL APS had significant effects on the ER dilation ultrastructure. There was a decrease in the ER dilation, and the widths of the ER lumen in HT29 cells when compared to that of the model group (*P*<0.05). These results suggest that the reduced expression of GRP78 and CHOP in HT29 cells, is potentially through inhibition of the activation of the PERK-eIF2a signaling pathway by APS.
